# Investigating Addiction in the Changing Universe

**DOI:** 10.5539/gjhs.v6n6p73

**Published:** 2014-07-15

**Authors:** Mojgan Dastoury, Tayebe Aminaee, Raheleh Ghaumi

**Affiliations:** 1 University of Socialwelfare and Rehabilitation, Center of Social Determinant on Health, Tehran, Iran; 2 Unit of Adolescent and Youth Health and School in Sanitation Center of Golestan Province, Iran

**Keywords:** addiction, globalization, social change, secondary analysis

## Abstract

The process of globalization as the most significant characteristic of modern era is facilitated by several factors including Information Technology, the industry of production and distribution of information, the flow of goods, services, human beings, capitals, information and etc. This phenomenon, along with the complex and various identities and life styles created by the national and transnational determinants, has widely changed the nature of social phenomena, including addition. The present study aims to investigate the contribution of sociological studies in the field of addiction during 2001 to 2011 in Iran. This is done through performing content analysis on 41 peer reviewed papers. The selected samples were surveyed and compared according to theoretical frameworks and the social groups under study. The results showed that the analyzed papers extensively overlooked the process of contemporary social changes in Iran which could be caused either by the theoretical basis of the studies or the social groups under study. Following the theoretical views of previous decades, these papers largely considered addiction as a type of social deviation and misbehavior related to the men living in urban areas.

## 1. Introduction

Giddens suggests that we should take a look at our local supermarket as the most far-fetched place for sociological studies in case we are interested in understanding the social phenomena (Giddens, 2007). This suggestion reminds sociologists of the necessity of addressing the changing nature of social phenomena and the significant roles they play in moving towards globalization. As Nash explains, this is a reality developed by the complex and interwoven economical, technological, cultural, environmental, and social processes as well as the increasing flow of products, capitals, information, population, and beliefs which might create increasing risks and conflicts among the nations as well (Nash, 2012).

In such a network universe (Castells, 2006), in which the time and space are compressed, the natural distances and borders have lost their conventional meaning and significance as a result of availability of information and communication technology. These conventional meanings are constantly changed and reconstructed as a result of various multi-dimensional and universal flows. The same as any other social phenomena, drug addiction also develops and emerges in different levels, aspects, and forms. Consequently, this phenomenon has been the focus of several scientific attempts and investigations for long and it has been explored from the standpoints of different knowledge domains.

Addiction is mainly defined either as a biological-psychological phenomenon for facilitating the gradual adaption of organism with some toxic materials (Orang, 1988) or as a chronic and relapsing disorder based on two elements of behavioral and physical dependence (Kaplan & Sadock, 2007). Nonetheless, some scholars believe that addiction should be considered at the first level as a social phenomenon and then a psychological and personality issue (Sedigh Sarvestani, 2005; 2007). Addiction has been investigated from different angles. For example, physicians have tries to examine various methods and medications to remove the toxics from the addicts’ bodies in order to recover their health; biologists have studied the biologic harms that drugs could bring about for the ecosystem; economics have focused on the financial aspects and economic losses that might result from addiction; and finally psychologists have investigated the characteristics and mental traits of addicts. However, the sociologists go beyond the individual person and consider the factors such as socio-cultural variables and structures, the life styles involved in increasing or decreasing the addiction and the potential effects and risks for individuals, families, and communities. The same as other societies, Iranian academic and scientific groups have been also concerned with this issue and the rich literature including peer reviewed articles and journalistic texts indicate to the serious attempts made.

Iran, as one of the countries with the highest level of drug abuse, bears various costs besides the health, psychological, social, political consequences caused by addiction. These costs and consequences are represented in the loss of young and working human resources, the loss of dynamic and creative resources, family breakdown, increasing rates of crime and social deviations, and huge costs for security issues by police forces. Therefore, producing precise and updated information and knowledge could play a supporting role in improving the relevant policy makings, plans, and actions taken to confront this problem. This necessitates descriptive evaluations and detailed analyses as well as constructive criticisms over the process of production, distribution, and implication of the obtained knowledge. In the same line, the present study considered this issue from a sociological perspective and aimed to investigate this question: “what is the share of sociological studies in knowledge production about addiction from 2001 to 2011?”

The significance of choosing the mentioned time span is that several research centers for addiction were founded in 2002 and as a result this issue took priority in scientific and research centers. Consequently, the studies on this topic achieved a considerable growth in the following decade. The present study aimed to probe and describe the present bulk of studies that covered the issue of addiction in the fields of sociology and psycho-sociology in the recent decade. For this purpose the theoretical, methodological, and regional aspects of the papers published in scientific journals in the mentioned decade were analyzed through content analysis. The results of such studies could lead to development of future studies and focusing on overlooked aspects and finally improving the research decision makings.

### 1.1 Theoretical Concepts: Addiction in Sociological Theories

A comprehensive examination over the history of contemporary human social life, regardless of the nature of society and the level of development, reveals that drug abuse attracted the attention of sociologists as a social issue when the consequences of urbanism and industrialization emerged in different forms. In such conditions, various scholars sought for making fundamental and theoretical assumptions about the social and cultural foundations of addiction in order to overcome the reductionism by biological and psychological theories. Instead of considering addiction as an illness or a personal issue that is independent from historical, social, and cultural contexts, they tried to decode the forces, elements, as well as historical, social and cultural contexts intervening in this phenomenon (Granfield & Cloud, 1999). In the present study addiction was defined as continuous in-taking of drugs or any intoxicant substance. Beside, in this study the most current sociological theories were considered regarding addiction.

Initially, addiction to drugs attracted the theoretical interests by sociologist as a kind of social deviation, but gradually it developed to an independent domain among other scientific issues and concerns. The professional journal dedicated to this issue, which was established in 1996, offers a comprehensible list of studies dedicated to this issue and mentions Problem Behavior Theory, Theory of Reasoned Action, Social Learning Theory, Social Control Theory, Self-derogation Theory, Integrated Delinquency Model, Social Development Theory, And Theory of Triadic Influence as the most dominant theories in explaining addiction in the field of sociology (Anderson, 1998). Other theories in the same field include Social Anomy Theory (Akers & Sellers, 2013; Cohen, 2007); Labeling Theory (Abadinsky, 2011); ideas by R. E. Park and Ernest Burgess within the tradition of urban sociology of Chicago School (Suchar, 1978); Theory of Shaw and McKay within the same theoretical framework (Bursik, Harold, & Grasmick, 1993; Veysey & Messner, 1999; Paulsen, Matthew, & Robinson, 2004); The Human Capital Theory (Chmidt et al., 2004). The Differential Association (Wrightsman, Nietzel, & Fortune, 1998; Salimi, 2006) and Conflict Theory (White & Haines, 2008) are considered as extensive attempts made with the aim of theorizing about addiction as a separate and independent classification from the general umbrella term of social deviation.

In the next generation of the theories about this issue, or the theory of addiction in modern era, this social phenomenon is explained as a cultural issue with an emphasis on macro-level variables. In this regard, it is reasoned that the life style concerns and the harmful tensions for health have been multiplied in developed and industrialized societies and addiction is one of the consequences of mass production in free markets. Accordingly, Alexander believes that addiction has a strong relationship with “dislocation” and follows extensive social changes along with the development of capitalism (Alexander, 2000). Norman Zinberg indicates to the structure of new opportunities for men in proletarian class which is attained due to elimination of social and natural inhibitors. He analyzes the increasing phenomenon of drug abuse as a means of overcoming mental tensions caused by the rapid changes and also dealing with the difficulties of labor and alienation (Alexander, 2000; 2008). According to this viewpoint, addiction is a type of adaption to the universal social conditions in which searching for meaning and individual identity is increasingly assigned to personal actions and attempts. Regarding the cultural policies related to addiction, David Forbes (1994) refers to the difficulties in satisfying the needs such as power, security, and self-expression, and claims that addiction is a political and cultural issue and as well as a compensatory mechanism. As Norman Denzin (1993, p. 8) states, addiction could even be considered as a representation of cultural failure from a social criticism perspective.

As Jenkins believes, different social minorities, including women and ethnic groups, are different in expressing “reflective self” against the external forces affecting identity. This ability is in fact determined by the level of availability to wealth as well as material and cultural resources (Jenkins, 2013). Giddens evaluates this issue from another aspect and indicates to the fact that traditions are fading away due to lack of self-control in many individual and social dimensions of life. He states that this is a consequence of several conditions including the increasing role of media in eliminating time and place restrictions and challenging personal and group identities through breaking the well-defined traditional and social frameworks as well as searching the modern elements at universal levels (Giddens, 2012; 2007; 2014). He adds that personal issues including addiction have taken a public aspect and addiction should be investigated and understaood in a society where traditions are fading away and searching for *self* is taking critical importance. In such conditions, addiction and dependence are considered as some ways for adapting with personal, fragmented and interrupted experiences in social life (Giddens, 2012).

But globalization theoreticians mainly focus on *meaning* and *identity* and emphasize changes in major functions of social institutions such as family, job, religion, education, and also basic changes in population, cultural interactions, technological aspects, and the decline of local markets and globalization of markets. Following that, there has been a flow of in-society risks and vulnerabilities and a feeling of “dislocation and detachment” (Fukuyama, 1995) which led to increasing attempts by the addiction theories to examine the concept of “dislocation” in the globalization era. The same as any other social phenomenon, addiction has emerged in completely different forms and aspects and the researchers in this field face difficulties due to the considerable varieties in tendencies, production and usage rates, the social status, age, gender of the users, the production and distribution networks, varieties of drugs specially the chemical types. This great diversity calls for several policies and strategies in order to treat and rehabilitate the abusers and fight against trading drugs.

Today, in many countries, particularly the developing nations, the interactive effects of gender (Graham & Zeballos, 1998; Room, 1996; Vega, 2002; Wingood & DiClemente, 2000; Moon et al., 1999), age, the supportive or resistive effects of ethnical identity and the relevant factors (Gazis et al., 2010; Losoya et al., 2008; Prado et al., 2008; Brook et al., 1998; James, Kim, & Armijo, 2000; Brook et al., 2006) and the potential risks of drug abuse are evaluated and examined extensively. This indicates that the researchers address the socio-cultural conditions of several social groups seriously. On the other side, these efforts highlight the active, independent, and interactive participation and role of these variables in the process of identity development and self-expression and the potential relationship between these variables and the risks of drug abuse.

Generally speaking, the massive changes in the patterns of drug abuse in the globalizing universe has caused the studies to shift their focus from investigating and theorizing addiction as a kind of social deviation to a personal choice with the aim of seeking pleasure, identity, and other individual aspects; from a behavioral issue of socially rejected individuals to a behavioral issue for the middle and high social classes; from a behavioral issue of men from proletariat class to a behavioral issue among the educated and prosperous women; and from a local concern to a universal one.

## 2. Methodology

The present study employed a cross-sectional/descriptive approach by using content analysis in order to analyze and investigate 42 peer reviewed articles related to the subject of addiction in the field of sociology and psycho-sociology. The selected papers were published during the time span of 2002-2011 and are available in Iranian academic data bases including SID, Magiran, Irandoc, and Noormags. As the first step, the mentioned websites were searched by using keywords such as addiction, addict, and drugs. This stage revealed that addiction has been explored in various disciplines such as medicines, pharmacology, psychology and behavioral sciences, criminology, law and police studies, social studies, and economics. In this step 512 papers were selected but after excluding the journalistic and the overlapping papers as well as those out of the mentioned time span, 42 papers were selected from the fields of sociology and psycho-sociology through random sampling. The obtained papers showed that that these two fields had a limited share in comparison to other fields, specifically medicines and psychology. Finally, the theoretical and methodological approaches, and the gender, age, ethnical, religious, and regional variables were coded in each paper and the information was categorized and later analyzed.

### 2.1 Limitations of the Study

During the research process there were some limitations that created several difficulties for the data collection phase. These limitations were caused by lack of access to coherent databases about the studies on drug abuse and drugs, lack of organization in journal and scientific papers, and lack of categorization of papers according to the scientific fields. Generally due to the interdisciplinary nature of addiction as a subject, the relevant papers were published in several fields such as biology, pharmacology, psychology and educational sciences, law, policy, and military sciences. Therefore, a considerable number of papers gathered in the first step were repeated or did not serve the purpose of the study. The final sample comprised of 42 papers which met the mentioned criteria. Finally, these papers from the fields of social psychology and sociology were analyzed and investigated to determine the contribution of these fields.

## 3. Results

The classifications of the results for the present study showed that a range of sociological theories were employed in the analyzed papers. These theories include Social Anomia, Social Labeling, Social Learning, Differential Association, Social Control, Socialization, Sub-cultures Deviants, Relative Deprivation, Unequal Opportunities and Social Deprivation Theory, Urban Ecology Theory, and Medicalization of Modern Society Theory. The results revealed that in these papers Social Anomia, Labeling and Social Control theories were used most frequently by the researchers. The results also indicated that Medicalization of Modern Society Theory, Bourdieu cognitive theory and Social Exclusion theories were applied less frequently than other theories. And the theories such as Human Capital, Social Capital and the Neo-Realism theories were not used in the analyzed papers. In addition, the results indicated that these theories were mainly mentioned as a type of review over a set of theories and the papers did not focused on evaluating and testing a specific theory in the specific circumstances of Iran society. In other words, in the corpus studied no particular theoretical framework was adopted for investigating the intervening factors in addiction, but a set of factors and causes were presented as the influential factors that were generally irrelevant to the applied theoretical framework. This gap between the resulting factors and presented theory was so considerable in some of the papers that it minimized the role that theory plays in the development of the study.

As far as the methodology is concerned, one of the most evident commonalities in the papers under study was that the researchers mainly tended to apply quantitative/survey method, questionnaire as instrument, and statistical techniques and tests for the analysis of the data.

According to the result of this study, a significant number of these papers used questionnaires as their instruments. From the analyzed papers, 9.5% used qualitative method and interview, observation, focused group discussions, and documental techniques as the instruments.

As the results indicate 26% of the papers investigated the adolescent, youth, and university-student age groups (Table 2).

**Table 1:** Frequency distribution of studies on addiction based onmethodology

Method of Research	Number of Articles	Percentage
Quantitative	31	73.5%
Qualitative	4	9.5%
Qualitative	7	17%
Quantitative		
Total	42	100%

**Table 2:** Frequency distribution of studies on addiction based on Age Groups

Age group	Number of articles	Percentage
Youth and adolescent	11	26%
Other Groups	27	64%
Theoretical discussion	4	10%
Total	42	100%

Among the papers 60% were related to the urban or province regions; 15% included country domain, and 7.5% focused on rural areas (Table 3).

**Table 3:** Frequency distribution of studies on addiction based on level of study

Level of study	Number of articles	Percentage
Urban	29	69%
Rural	2	5%
National	6	14%
Theoretical discussion	5	12%
Total	42	100%

Only 7% focused on females and about 70% of the studies investigated addiction in both genders (Table 4).

**Table 4:** Frequency distribution of studies on addiction based on gender of Samples

Gender	Number of articles	Percentage
Female	3	7%
Male	9	21.5%
Both	30	69.5%
Total	42	100%

As the Table 5 shows, 5% studied this issue among the religious/ethnical minorities.

**Table 5:** Frequency distribution of studies on addiction based on social group of samples

Social Group of Samples	Number of articles	Percentage
Minorities	2	5%
Majorities	40	95%
Total	42	100%

## 4. Discussion and Conclusion

Studying the reality of human social life in the globalization era seems to be a necessity due to current conditions that facilitate the social interactions beyond the traditional place and time limitations. The non-stop and continuous flow of information, ideas, goods, services, and life styles followed the development of information technologies have changed the universe into, as Mcloham claims, a village along with the fourth phase of industrial revolution. This has led to multi-culture, plurality and variety of identity sources, changes, permutations, or alterations in some of the social structures and an increase in the diversity of life styles and personal choices for individuals in this universe. These factors together establish a novel individualism with an inevitable connection to constant and dynamic structuring and restructuring of self-identification, shrinkage of effects and extent of tradition and values involved in goal setting and the social actions as well as means to attain the goals due to local-universal interactions, the changing, unstableness, flexibility, and variety of the phenomena.

In a couple of decades ago the researchers mainly focused on the several socio-cultural consequences of industrialization and urbanization in modern societies. This was done with the aim of identifying and explaining the causes of social deviations including addiction. Nevertheless, today there have been some new conditions that could not be overlooked by the addiction researchers. These conditions include the expansion of socio-cultural borders of rural areas; further interactions with urban areas as a result of development of the villages to prevent immigrations; improving the transportation facilities for villages; the increasing effects of media including radio, television, satellite, and Internet in rural areas specifically among youths; and the increasing commonalities of urban and rural life styles. In other words, in the present conditions the involving factors such as self-alienation, anonymity, social distances, increasing expectations and tensions, and etc. could not be considered as the urban phenomena in explaining the high rate of deviations and crimes. The villagers also could not be supposed as the immigrating groups who turn into different social deviations, including addiction as a result of experiencing value conflicts and failing to manage the value and identity crisis when facing the new and unfamiliar conditions in cities.

In spite of the changes in the conditions, investigating the literature on addiction in the recent decade shows that the studies have constantly taken the same approaches to examining addiction. They commonly focus on urban areas as the center of social deviation and crimes. As the results of the present study revealed, only 5% of the analyzed papers focused on different aspects of addiction in rural regions. It shows a disproportionate attempt by the addiction researchers to take the rapid process of social changes into account.

Considering the age as a variable, the results of this study indicated that there is an obvious gap between research interests and the existing changing state of social conditions in Iran. The theoretical approaches regarding addiction show that addiction, which might be regarded as a social deviation, an attempt to gain social identity, a reaction to the inequalities of modern life, or merely a personal choice of lifestyle, is largely considered as an issue related to youths and adolescents rather than the old and middle-aged people who have reached higher stability in personal, social, and cultural states (Winick, 2013; Gravett, 1999; Amiri et al., 2014). While only 28% share of papers is dedicated to the youths, this group experiences the highest level of interaction with information networks in educational, job, and entertainment fields which exposes this group to the thoughts and lifestyles beyond the national borders. Therefore, they receive the highest amount of effects from the globalization phenomenon (Rabiei & Shahghasemi, 2009). In addition, this age group mainly orients their behaviors to future, satisfying the independency and seeking identity developing needs, youth energy, modernity, the peer group (Azad Armaki, 2007), and criticizing the dominant cultural values. Consequently, it could be expected that adolescents and youths are in higher levels of danger for drug abuse in comparison to other age groups. Furthermore, the drug abuse patterns among youths indicate specific features in comparison to the older age groups in which addiction is considered as a fixed behavioral pattern (Winick, 2013; Gravett, 1999; Amiri et al., 2014). These specific features necessitate specialized and further research and investigation. Unlike old-age groups who use the drug in an under-control manner and for medical purposes, young and adolescent groups use drugs recklessly and with no control. In fact, the young and adolescent age groups mainly use drugs for the purposes of detachment from time and place and its compulsiveness and also experiencing unknown states of hallucination. These groups tend to use chemical and new types of psycho-stimulant drugs such as methamphetamine (commonly referred to as glass or crystal) rather than the conventional types such as opium and heroin (Amiri et al., 2014).

The concept of cultural *dislocation*, which results from wide-range and rapid social changes, disappearance of values and common life styles, and the ongoing replacement of new values and life styles as well as *compulsiveness*. Giddens believes that dislocation plays a considerable role in constructing individual identities and life conditions due to replacement of restricted social traditions by modern elements. This indicates to a research necessity for focusing on different social groups other than the age groups. The reason is that the roles and status of individuals and groups in this changing world reveal, on the one side, the diversity and plurality of identity-giving sources and, on the other, a resistance for expressing self-reflection. In fact, the resistance of modern human being to institutional categorizations and classifications as well as *authoritatively allocation of identity* is not an objection; rather it is an attempt for exposing individual’s dynamic actions in constructing his/her reflective identity. As Jenkins argues, the resistance of minority groups including women, ethnical, and religious groups to social and institutional ranking could represent a change in status and availability to novel identity-giving sources. Therefore, addiction either as an active and resistive reaction as an objection or passive one as a compensatory mechanism could be representation of reactions by social minorities against their subordinate status in *cultural division of labor* as well as lack of justice in life chances for achieving an equal status and a self-constructed and independent identity. But the 5% share dedicated to minority groups by studies conducted on addiction in the recent decade might be considered as a sign of ignorance toward existing social necessities. This ignorance is also represented in the lack of implications from research by the government. In fact, the implicational aspects of the studies and papers in the field are rarely considered in the socio-cultural plans and policy making attempts by the government.

In general, the results of the present study point to a new trend of efforts for investigating various aspects of this subject by the main institutions for the process of developing major socio-cultural programs and policy makings. These results also show an overlook of updated sociology knowledge in investigating the new circumstances in Iranian society. As it was discussed, most of the theories applied as the theoretical framework for the papers, following the traditional assumptions, considered addiction as a socio-cultural norm-breaking behavior and they failed to attend to the features specific to each age, gender, ethnicity, religion, social class, and region. The papers under study did not consider addiction as a response to the socio-cultural conditions of such a society which is experiencing modernization in different dimensions and levels. In fact, addiction was not taken as an attempt to reveal self-reflection in an era that inconsistency, *compulsiveness*, fluidity, flexibility, insecurity have dramatically changed the social phenomena, including addiction. These conditions are, on the one hand, caused by the rapid and wide-range changes that result from information technologies around the universe and, on the other hand, by the resistance from local and traditional structures toward revision through maintaining the imperative social arrangements and relationships with the aim of controlling and supervising the citizens’ behaviors. Therefore, the research literature from the recent decade might not provide the policy making actions and governmental institutions with reliable and applicable solutions to the problem of addiction. The reasons to be mentioned for this claim include overlooking the new circumstances and the relevant necessities, ignoring the updated sociology knowledge, applying and reapplying scientific theories which do not address the existing needs.
